# Mechanical and Thermal Performance of In-Situ Synthesized PDMS-SiO_2_ Composite as Electrical Insulating Coatings

**DOI:** 10.3390/molecules30102107

**Published:** 2025-05-09

**Authors:** Aldo Cordoba, Rossana Faride Vargas-Coronado, Rodrigo Velázquez-Castillo, Juan Valerio Cauich-Rodríguez, Karen Esquivel

**Affiliations:** 1 Graduate and Research Division, Engineering Faculty, Universidad Autónoma de Querétaro, Cerro de las Campanas, Santiago de Querétaro 76010, Querétaro, Mexico; acordoba07@alumnos.uaq.mx (A.C.); rodrigo.velazquez@uaq.mx (R.V.-C.); 2 Centro de Investigación Científica de Yucatán, Unidad de Materiales, C. 43 No. 130 x 32 y 34, Col. Chuburná de Hidalgo, Mérida 97205, Yucatán, Mexico; ross@cicy.mx

**Keywords:** in situ sol–gel, hybrid O/I systems, wettability behavior, thermal behavior, mechanical properties

## Abstract

Polydimethylsiloxane (PDMS) has been extensively employed in electrical insulation applications owing to its excellent thermal stability, hydrophobicity, and dielectric properties. However, its inherent mechanical limitations require structural reinforcement to enhance its performance under more demanding operational conditions. In this study, the mechanical, thermal, and surface properties of PDMS-SiO_2_ nanocomposites synthesized via in situ sol–gel process was systematically investigated. The influence of different SiO_2_ nanoparticle concentrations (5, 10, and 15 wt%), sol–gel catalyst type (acidic and alkaline), and tetraethyl orthosilicate (TEOS) crosslinking agent ratios (15:1, 10:1, 5:1) was evaluated. Tensile mechanical testing, dynamic mechanical analysis (DMA), and thermogravimetric analysis (TGA) revealed that the incorporation of SiO_2_ notably improved both the mechanical strength and thermal stability of the composites. The 5-15b and 10-15a composites exhibited the highest tensile stress and viscoelastic modulus among all samples. Furthermore, the composites retained key functional properties, including hydrophobicity, high volumetric electrical resistivity (~10^11^ Ω·cm), and strong adhesion. These findings confirm the potential of in situ PDMS-SiO_2_ nanocomposites for use as high-performance insulating coatings in advanced electrical applications.

## 1. Introduction

Polydimethylsiloxane (PDMS) is a widely used polymer in various applications due to its remarkable flexibility, chemical stability, hydrophobicity, and thermal resistance [1]. Its high electrical insulation and chemical stability against environmental factors such as radiation and humidity make it an ideal candidate for protective coatings in electronic and electrical applications, including high-voltage cables and insulators in power plants [2]. However, PDMS exhibits limitations such as low mechanical strength and hardness, which requires its integration with additional materials to enhance its durability under external conditions and extend its operational life [3].

Several reinforcement strategies have been proposed to enhance the mechanical performance of PDMS when applied as an insulating coating. Among the most extensively studied are nanostructures based on metal oxides such as titanium dioxide (TiO_2_) [4,5], alumina (Al_2_O_3_) [6], and silicon dioxide (SiO_2_) [7,8], as well as carbon-based nanomaterials including carbon nanotubes (CNTs) [9] and graphene [10]. Previous investigations have demonstrated that TiO_2_ nanoparticles can enhance the elastic modulus and tensile strength of PDMS matrices; however, significant agglomeration tendencies and reduced flexibility have been reported, limiting their applicability [11]. Similarly, CNT-reinforced PDMS composites exhibit substantial improvements in mechanical strength and electrical conductivity; nevertheless, dispersion difficulties and the tendency to create localized stress concentrations negatively impact its tensile strength [12].

Nanocomposite materials based on PDMS and SiO_2_ have attracted considerable attention due to their improved mechanical and thermal properties [4,13]. The incorporation of SiO_2_ into the PDMS matrix did not alter the chemical environment or the spatial arrangement of the polymeric network, owing to the chemical similarity between the matrix and the reinforcement [7]. This compatibility enabled the preservation of the elastomeric behavior of PDMS and facilitated an effective dispersion of the reinforcing phase within the composite structure.

Despite the multiple nanostructures that have been shown to modify and improve the mechanical properties of these polymers, traditional approaches for the fabrication of PDMS composites have primarily relied on ex situ synthesis (pre-formed nanoparticles were dispersed into the polymer matrix) which ends up limiting the addition of reinforcers due to the difficulty of dispersion and agglomeration in high-viscosity polymers [14]. That is why the use of high-energy mixing techniques to achieve adequate dispersion is required [15], thereby limiting the maximum concentration of reinforcements that could be effectively incorporated. These conditions have ultimately led to suboptimal interfacial interactions between the polymer matrix and the nanoparticles, adversely affecting the final performance of the composite materials [9].

To address these limitations, recent research efforts have been focused on the development of in situ synthesis techniques, which allowed the formation of nanoparticles directly within the polymeric matrix [14]. In situ synthesis methods include emulsion polymerization [16], hydrothermal-assisted synthesis [17], and the sol–gel process [7]. Among these, the sol–gel method has offered several advantages, including enhanced nanoparticle dispersion, adaptability to diverse polymer matrix synthesis conditions, and tunability of the physicochemical properties of the reinforcements by modifying key synthesis parameters such as catalyst type, pH, and precursor concentration [18]. For SiO_2_ nanoparticles, the influence of sol–gel catalysts was found to be a critical factor in determining the size, morphology, and surface chemistry of the synthesized nanoparticles, thereby potentially impacting the overall performance of the resulting PDMS composites [19].

Although significant advances have been achieved in the development of PDMS–SiO_2_ composites [7], comprehensive investigations addressing the influence of key synthesis variables—such as nanoparticle concentration, sol–gel catalyst selection, and crosslinking degree—on the mechanical and thermal performance of these materials remain limited. Previous studies have indicated that increasing nanoparticle content can improve the mechanical reinforcement of PDMS; however, excessive nanoparticle loading has been associated with agglomeration phenomena, which may detrimentally affect the uniformity and performance of the composite material [4,20]. Furthermore, the effect of sol–gel catalyst conditions on the structural homogeneity and interfacial interactions within the PDMS matrix has not yet been fully elucidated and continues to be the subject of ongoing research efforts.

Previous studies have emphasized the importance of combining crosslinking processes and nanoparticle incorporation; however, the competitive nature of the nanoparticle synthesis and polymer crosslinking reactions often limited the extent of polymer–nanoparticle interaction and, consequently, the final properties of the composites [21]. Additionally, although higher degrees of crosslinking generally enhanced the mechanical strength and thermal stability of PDMS-based systems, they also tended to induce excessive rigidity and reduce flexibility, which could negatively affect the performance of the material when applied as a coating [22].

In addition to the enhancement of mechanical properties, attributes such as hydrophobicity, electrical insulation, and adhesion are crucial for the optimal performance of these composites when employed as insulating coatings [1,3].

Hydrophobicity and superficial adhesion play a critical role in the durability and protective capability of insulating coatings, as they help prevent moisture absorption, contamination, and delamination which degrade electrical insulation [23]. Similarly, maintaining electrical insulation properties is essential to ensure the suitability of these composites for electrical applications [24]. The formation of composites through the incorporation of SiO_2_ reinforcements and variations in crosslinking conditions may impact these properties and warrants further investigation.

This study continues the evaluation of the in situ synthesis approach for PDMS–SiO_2_ composites previously developed by the research group. In earlier work, the physicochemical characterization of sol–gel-synthesized SiO_2_ nanoparticles and their corresponding composites was conducted through FTIR, Raman, XRD, and XPS analyses. The present investigation expands upon these findings by examining the influence of synthesis parameters—specifically nanoparticle concentration, sol–gel catalyst type (acidic and alkaline), and crosslinking agent (TEOS) ratio—on the mechanical and thermal properties of the resulting composites. The objective is to gain a deeper understanding of how these variables modify the internal chain mobility mechanisms within the polymer matrix. The results contribute to the advancement of polymer nanocomposites for electrical insulation applications and may serve as a foundation for future developments in nanoreinforced functional coating materials.

This study continues the evaluation of the in situ synthesis approach for PDMS–SiO_2_ composites previously developed by the research group. In earlier work, the physicochemical characterization of sol–gel-synthesized SiO_2_ nanoparticles and their corresponding composites was conducted through FTIR, Raman, XRD, and XPS analyses [7]. The present investigation expands upon these findings by examining the influence of synthesis parameters (nanoparticle concentration, sol–gel catalyst type (acidic and alkaline), and crosslinking agent ratio) on the mechanical and thermal properties of the resulting composites. The objective is to gain a deeper understanding of how these variables modify the internal chain mobility mechanisms within the polymer matrix. The results contribute to the advancement of polymer nanocomposites for electrical insulation applications and may serve as a foundation for future developments in nanoreinforced functional coating materials.

## 2. Results

To guarantee the accurate identification and analysis of the different study groups, a specific nomenclature was established. Each composite was designated by first indicating the PDMS:TEOS weight ratio (15, 10, or 5), followed by the SiO_2_ reinforcement content (0, 5, 10, or 15 wt%), and finally by the synthesis medium, with “a” denoting acidic conditions and “b” representing alkaline conditions. For instance, the composite labeled 5-15b corresponds to a PDMS:TEOS weight ratio of 5:1, reinforced with 15 wt% of SiO_2_ nanoparticles synthesized under alkaline conditions.

### 2.1. PDMS-%SiO_2_ Mechanical and Thermal Properties

#### 2.1.1. Tensile Mechanical Analysis

**Figure 1:**
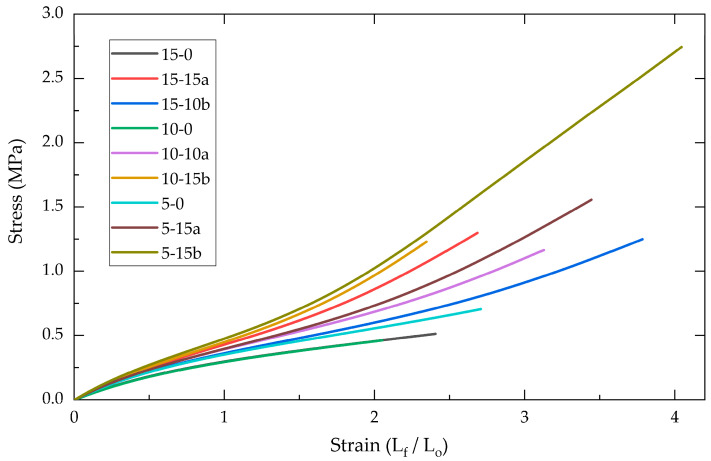
Stress–strain profiles of PDMS-%SiO_2_ composites for SiO_2_-a and SiO_2_-b reinforcers.

Tensile tests were conducted on the various PDMS-%SiO_2_ polymeric composites to determine the changes in mechanical behavior caused by different concentrations of SiO_2_ reinforcer (0%, 5%, 10%, and 15%) obtained via sol–gel synthesis in acidic (a) and alkaline (b) media. In Figure 1, the stress–strain profiles for the different study groups are presented, revealing a notable increase in both ultimate tensile stress and strain compared to the control unreinforced polymers (15-0, 10-0, and 5-0). In the unreinforced polymers, two primary processes were observed—one occurring between 0 and 0.4 MPa and another above 0.4 MPa—which corresponded to the elastic and plastic deformation stages, respectively [1].

In the reinforced composites (PDMS-%SiO_2_), a third creep process was observed at 1.5 MPa, which is attributed to a plastic deformation mechanism commonly encountered in reinforced polymers and related to matrix–particle interactions [7,11].

**Figure 2:**
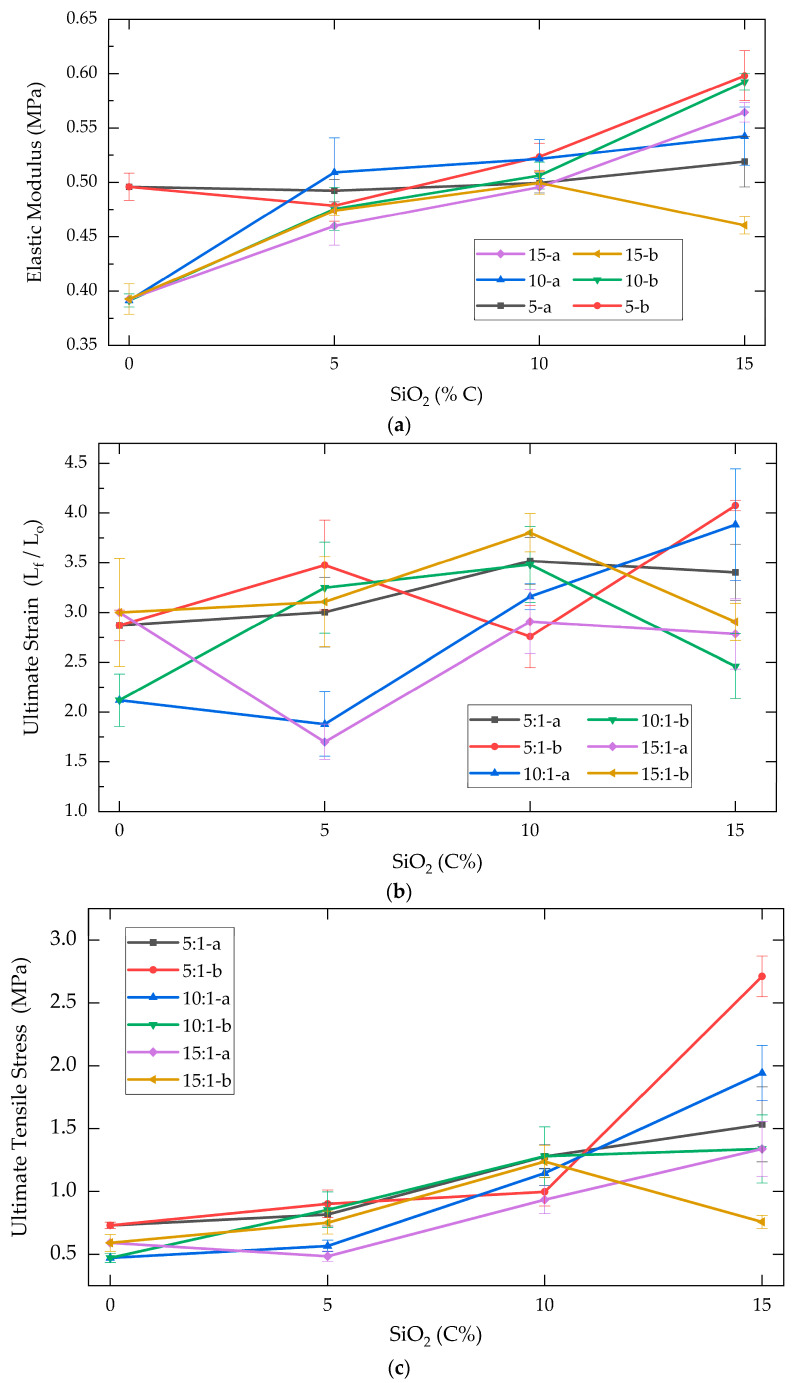
Mechanical properties of PDMS-%SiO_2_ composites: (**a**) elastic modulus, (**b**) ultimate deformation, and (**c**) ultimate tensile stress.

Based on the stress–strain profiles, the elastic modulus, ultimate strain, and fracture stress for each reinforced composite were calculated and presented in Table 1. In Figure 2, a graphical representation of the progressive changes in these mechanical properties (*y*-axis) is provided as the reinforcement concentration increased from 0% to 15% (*x*-axis).

For the unreinforced materials (15-0, 10-0, and 5-0), the elastic modulus increased with the PDMS:TEOS weight ratio, ranging from 0.393 to 0.496 MPa, corresponding to a 26% increase for the control group with the highest crosslinking concentration (5-0) [25]. Among the reinforced composite groups, the elastic modulus exhibited a positive correlation with the concentration of SiO_2_ reinforcement. The composites exhibiting the highest elastic modulus values were 5-15b and 10-15b, with values of 0.60 ± 0.02 and 0.59 ± 0.01 MPa, respectively. These values represented increases of 20% and 51% relative to its unreinforced control groups (5-0 and 10-0).

In contrast to the elastic modulus, the ultimate strain of the composites did not exhibit a discernible relationship with the SiO_2_ concentration. Within the unreinforced polymer samples, the 10-0 group exhibited the lowest strain at fracture, with a dimension ratio (D_f_/D_0_) of 2.12 (corresponding to a deformation of 212%). Among the reinforced composites, the highest dimension ratios were observed in 5-15b and 10-15a groups, with ratios of 4.08 and 3.88, respectively, representing increases of 42% and 83% compared to the unreinforced polymers.

The ultimate tensile stress property exhibited a rising trend as the concentration of SiO_2_ reinforcements increased. The unreinforced PDMS composites demonstrated fracture stress values ranging from 0.471 to 0.731 MPa, with the 10-0 group displaying the lowest fracture resistance.

For composites with low SiO_2_ concentrations (5a and 5b), fracture stress values ranged from 0.484 to 0.901 MPa. Gradually, the fracture stress increased from 0.934 to 1.281 MPa for composites with a 10% reinforcement concentration (10a and 10b, respectively). At the highest reinforcement concentration (15% SiO_2_), most composites exhibited a considerable increase in ultimate tensile stress, thereby differing from the behavior at lower concentrations [26]. The composites with the highest fracture stress for each reinforcer (SiO_2_-a and SiO_2_-b) were 10-15a and 5-15b (1.94 and 2.71 MPa, respectively). This value represented an increase of 371% and 412% in comparison with its control groups.

#### 2.1.2. Dynamic Mechanic Analysis

In addition to the tensile tests, dynamic mechanical analyses were conducted to elucidate the mechanical behavior of the various composites by examining variations in their viscoelastic properties and thermal transitions over a temperature range [27]. DMA tests were performed on the composites within a temperature window from−130 to 80 °C. Table 2 presents the thermal transition processes, including the glass transition temperature (T_g_), as well as the storage modulus (G′), loss modulus (G″), and tan δ (G′/G″) measured at 30 °C for each study group.

For all composite groups, a viscoelastic behavior characteristic of the PDMS polymer matrix was observed, with a larger contribution from the solid elastic response (G′) behavior after the crosslinking process [28]. A gradual increase in the storage modulus was observed within the incorporation of SiO_2_ nanoparticles. The composites that exhibit the highest reinforcement concentrations were 15-15a, 10-15b, and 5-15b.

Glass transition temperature of composites varied as a function of the crosslinking degree and reinforcement concentration, presenting the composites 15-15a, 10-15b, and 5-15b the highest transition temperatures. Composite 5-15b exhibited the most pronounced temperature increase (−52.54 °C), representing an increase of over 9 °C relative to the control polymer 5-0 (−61.77 °C). This behavior can be attributed to changes in the crosslinking degree, reducing the mobility of the polymer chains [1].

#### 2.1.3. Thermogravimetric Analysis

Once the mechanical tests (static and dynamic) had been performed, it was determined that the composites exhibiting the highest mechanical strength for each type of SiO_2_ reinforcement were 10-15a and 5-15b. Analyses of thermal properties of these composites were performed for a more comprehensive understanding of the interaction between the polymeric system and the selected variables (sol–gel catalyst, SiO_2_ concentration, and crosslinking agent concentration) on the thermal mobility of the polymer chains and thermal stability [29]. To identify these progressive changes in the thermal behavior of the composites with superior mechanical performance (10-15a and 5-15b), thermogravimetric analyses were performed.

**Figure 3:**
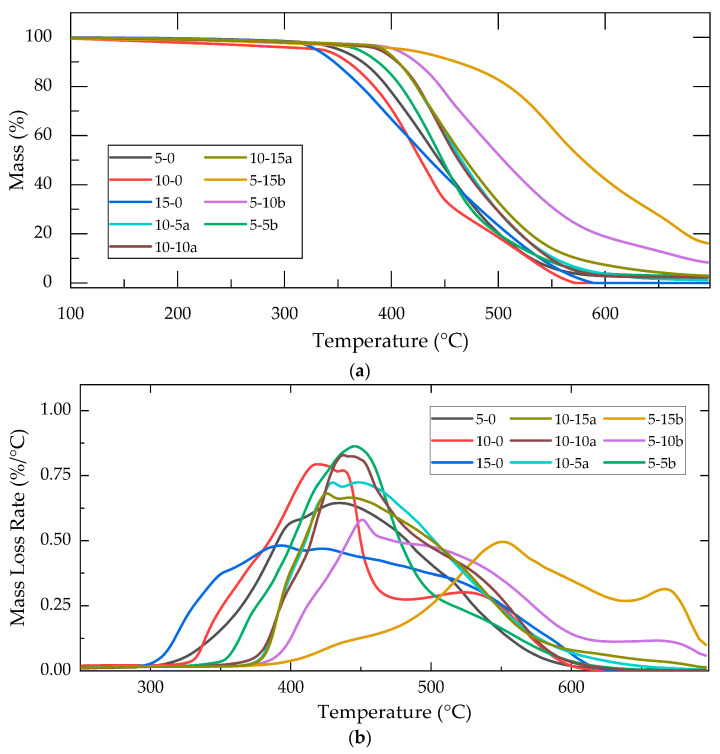
TGA thermograms of PDMS-%SiO_2_ composites: (**a**) weight loss percentage and (**b**) derivative TGA (%/°C).

Figure 3a shows the weight loss profiles of the best mechanical composite subgroups, from the control groups 10-0 and 5-0 up to the highest concentrations, 10-15a and 5-15b. In Figure 3b, the weight loss rate of the composites, as the temperature increased to 700 °C, is represented.

For direct comparison among the study groups, the onset of the primary degradation process was determined via linear fitting. The results for the different composites are presented in Table 3. Additionally, the weight loss rate, and percentage of residual mass after 700 °C were recorded.

From mass loss rate values shown in Figure 2b, two principal degradation processes were identified across the temperature window of 300–700 °C for all groups. These degradation processes were attributed to the rupture of the crosslinking chains and the subsequent degradation of the primary PDMS chains [11].

An increase in the onset degradation temperature was observed in the control groups (15-0, 10-0, and 5-0) as the crosslinker concentration change; as the amount of TEOS increased, the degradation onset temperature rose from 327 to 358 °C. This behavior was attributed to the higher crosslinking degree; a more extensively crosslinked matrix requires greater energy for polymer chain mobility, resulting in higher degradation temperatures [30].

For the reinforced composites, variations in thermal behavior were observed upon employing different SiO_2_ concentrations (0, 5, 10, and 15%) and sol–gel catalysts (SiO_2_-a or SiO_2_-b). Composites reinforced with SiO_2_-a exhibited a slight increase in the onset degradation temperature relative to the unreinforced polymer, reaching up to 395 °C (a 12% increase relative to 10-0), without an apparent dependence on reinforcement concentration. Conversely, composites containing SiO_2_-b particles demonstrated a gradual increase in the onset degradation temperature as the concentration of SiO_2_-b reinforcement increased, rising from 377 °C (5-5b) to 447 °C (5-15b), representing an increase of up to 25% compared to the control polymer (5-0).

In Table 3, the percentage of residual weight following heating to 700 °C was also presented. For 10-b and 5-a groups, a gradual increase in the composite’s residual weight was observed as the reinforcement concentration increased [10]. For 10-0 composition, zero residual mass was observed at 700 C; however, the polymer’s residual mass gradually increased with SiO_2_-a concentration, reaching 2.94 w% for 10-15a. Similarly, in the 5-0, a residual weight of 2.27% was recorded, which gradually increased with rising SiO_2_-b concentration, reaching 16.16% for the composite with the highest reinforcement concentration (5-15b).

The higher residual mass observed in composites synthesized under alkaline conditions (SiO_2_-b) compared to those synthesized under acidic conditions (SiO_2_-a) can be attributed to the enhanced crosslinking degree promoted in these systems. As previously reported, alkaline sol–gel synthesis routes favor the retention of higher concentrations of unreacted TEOS and other precursors after sol–gel particle formation [7], which subsequently can participate in additional crosslinking reactions within the PDMS matrix. This increase in crosslinking degree contributes to improved thermal stability and superior mechanical properties, resulting from the synergistic effect of increased crosslinking and the incorporation of reinforcing nanoparticles.

### 2.2. PDMS-%SiO_2_ Coating Characterization

Crosslinking efficiency, hydrophobicity, conductivity, and adhesion tests were conducted on composite subgroups selected from the mechanical and thermal analyses (5-15b and 10-15a). These evaluations were performed to assess the impact of the selected variables on the essential properties required for the application of these materials as insulating coatings [24].

#### 2.2.1. Crosslinking Efficiency

Crosslinking efficiency of selected composites was estimated via gravimetric weight loss tests, determining if high reinforcement concentrations significantly altered the crosslinking yield of PDMS. Triplicate composite samples were collected and weighed in their dry state (P_i_), following immersion in ethanol (P_h_), and after the drying process (P_S_). Using Equations (1)–(3), the solvent absorption percentage (A%), weight loss percentage of the coating due to the solvent (L_w_), and the crosslinking efficiency (X_y_) were calculated for the composites after removal of the remaining subproducts and unpolymerized chains. The results are presented in Table 4.

(1) $$A=\frac{P_{h}-P_{i}}{P_{s}}$$

(2) $$L_{w}=\frac{P_{i}-P_{s}}{P_{i}}$$

(3) $$X_{y}=\frac{P_{s}}{P_{i}}$$

Solvent absorption (*A*) in the various control composites exhibited a dependence on the PDMS:TEOS ratio, progressively decreasing from 3.65% to 2.23% as the crosslinker concentration increased. This reduced solvent absorption can be attributed to the concentration of the elastomer’s crosslinking agent [30]. In composites 10-15a and 5-15b, a clear reduction in solvent absorption was observed relative to the control polymers, with values reaching less than half those of the unreinforced samples, with 1.12% and 0.98%, respectively.

The crosslinking efficiency (X_y_), complementary to L_w_, exhibited similar trends across the various composites, with all study groups falling within a range of 96.4–98.3%. Nonetheless, in the control groups, a higher concentration of TEOS crosslinker was associated with a slight increase in crosslinking efficiency, attributed to the presence of un-crosslinked polymer chains that were displaced by dissolvent [31]. Conversely, the reinforced groups exhibited less efficiency than those of the control groups, a trend attributed to a higher concentration of by-products generated during hydrolysis and condensation reactions, as previously demonstrated [7]. These by-products were displaced and eliminated from composites during the solvent absorption and drying processes.

#### 2.2.2. Hydrophobicity and Electric Insulation

**Figure 4:**
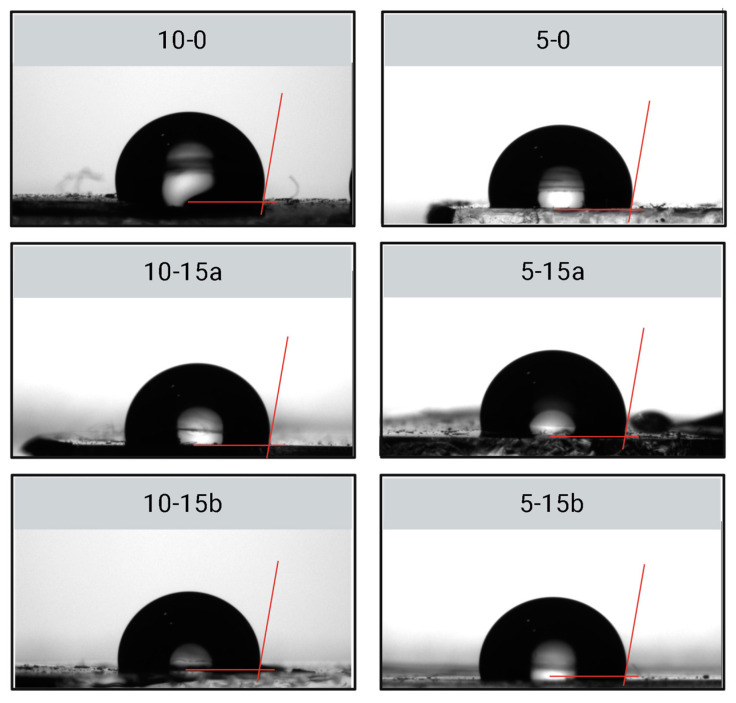
Water droplets on PDMS-SiO_2_ selected composites during contact angle testing.

Hydrophobicity of composites was assessed by measuring the water contact angle in air on the surfaces of the selected PDMS composites (10-15a and 5-15b) as well as on control polymers. Images obtained from the contact angle tests are presented in Figure 4, and the measured angles in Table 5.

No significant change in water contact angle (neither increase nor decrease) was observed in the reinforced composites (10-15a and 5-15b) compared with the unreinforced polymers (10-1 and 5-0). All selected composites exhibited a contact angle greater than 95°, thereby confirming their hydrophobic nature and falling within the normal PDMS contact angle range (95° to 113°) [25]. These results were attributed to the tendency of PDMS to migrate at the surface upon deposition, leading to the formation of a superficial layer predominantly composed of PDMS chains [32].

In addition to their hydrophobic properties, the preservation of the electrical insulation of PDMS coatings is deemed essential for their application in the electrical industry. The conductivity of the composites with superior mechanical performance and higher reinforcement concentrations (10-15a and 5-15b) was evaluated. The volumetric resistivities were determined via conductivity testing using the reversed voltage method [33] and recorded in Table 5.

The volumetric resistivity values obtained for both the reinforced composites (10-15a and 5-15b) and the control groups (10-0 and 5-0) fell within a comparable range, with all samples exhibiting a volumetric resistivity of approximately 10^11^ Ω/cm, without significant differences. These values were consistent with the PDMS reported properties for insulating composites indicating that the addition of SiO_2_ reinforcements up to a concentration of 15% neither reduced nor increased the electrical resistivity of these composites [34].

#### 2.2.3. Superficial Adherence

**Figure 5:**
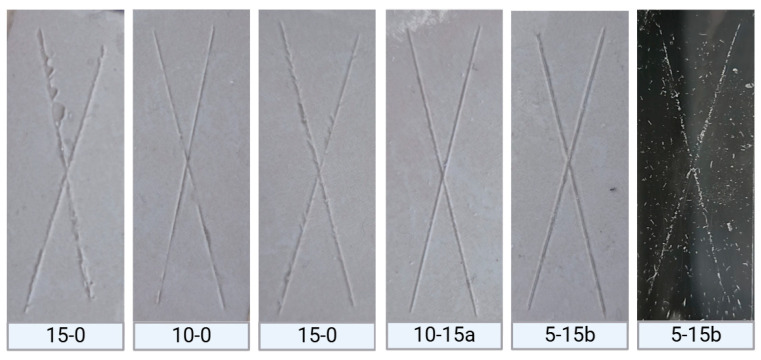
Adhesion test marks following ASTM D3359 on the surfaces of PDMS-%SiO_2_ composites.

In addition to the surface properties of the composites, maintaining the adhesion of PDMS to ceramic and polymeric substrates is crucial for its application as insulating coatings. Figure 5 presents the results obtained from adhesion tests conducted using the cross-cut tape method, following the ASTM D3359 standard [35].

Composites were analyzed according to the ASTM D3359 standard based on the amount of material removed during testing, with six possible categories (A0–A5). The reinforced composites (10-15a and 5-15b) and their respective control groups (10-0 and 5-0) were classified as A4, indicating the formation of cross-cut marks on the tape after testing.

In contrast, the 15-0 control group was classified as A3, suggesting variation in the adhesion level. This variation appears to be influenced by the crosslinking degree of the PDMS matrix, showing a behavior contrary to that reported for other polymer systems [36].

These classifications are considered indicative of good surface adhesion and align with values reported in previous papers [4]. The adhesion tests demonstrated that the presence of SiO_2_ nanoparticles (SiO_2_-a and SiO_2_-b) within the polymer matrix did not significantly affect the adhesion properties under the ASTM D3359 standard.

## 3. Discussion

This investigation demonstrated significant changes in the mechanical and thermal behavior of PDMS composites due to modifications in the selected independent variables, including crosslinking agent concentration (TEOS), sol–gel catalyst type, and SiO_2_ nanoparticle concentration. These results indicate potential alterations in the chain mobility processes of the polymer, directly influencing the composite’s structural characteristics and mechanical performance [37].

During tensile mechanical testing, the crosslinked PDMS chains experience an initial elastic unfolding process (elastic deformation), transitioning from a coiled to an extended spatial conformation. As deformation continued, the rupture of crosslinking bonds (plastic deformation) is initiated [1]. Similarly, during thermal analyses (DMA and TGA), the temperature elevates the vibrational energy of the molecules, progressively enhancing their mobility until the rupture of the crosslinking bonds (~430 °C) occurs, followed by the breakdown of the main polymer chains (~530 °C) [11,28]. These transitions also reflect an improvement in the material’s thermal stability, evidenced by elevated degradation of onset temperatures in reinforced systems. Such enhancements in physical properties contrast with the relatively minor changes observed in the chemical structure of the composites, as reported in previous studies [7].

When compared to other PDMS-based composites reported in the literature, the SiO_2_-reinforced systems developed in this study exhibited competitive mechanical properties. For instance, PDMS composites reinforced with TiO_2_ nanoparticles have reported tensile strengths ranging from approximately 1.6 to 2.8 MPa at loadings between 2.5 and 15 wt%, although presenting a limited elongation capacity [11]. Similarly, CNT-enhanced PDMS composites have achieved tensile strengths between 0.5 and 1.7 MPa at concentrations ranging from 2 to 10 wt%, while largely maintaining the original elongation behavior of PDMS [12]. In contrast, the SiO_2_-reinforced composites synthesized herein demonstrated tensile strengths in the range of 2.3–2.6 MPa (Table 1), comparable to TiO_2_-based composites, but notably surpassing the deformation capabilities reported for CNT-based composites, all while preserving essential properties such as hydrophobicity and electrical insulation.

The control groups (15-0, 10-0, and 5-0) demonstrated that an increased TEOS concentration led to a significant enhancement in mechanical behavior of the PDMS matrix. This behavior was attributed to an increase in the crosslinking degree within the matrix [30]. As the TEOS concentration increased, the number of crosslinking bonds also rose, restricting the mobility and subsequent sliding of polymer chains. This effect has been particularly evident in mechanical properties such as the elastic modulus [38]. Previous studies conducted by the research group have corroborated this behavior, obtaining a composite with increased stiffness and mechanical strength [4].

Previous investigations have demonstrated that the incorporation of nanostructured reinforcements within a polymeric matrix such as PDMS modifies the internal organization of the polymer chains, resulting not merely in the formation of a two-phase system (matrix–reinforcement) but in a material exhibiting active internal interactions [39]. Unlike micrometer-scale reinforcements, the presence of nanoparticles enables direct interaction between the long polymer chains and the reinforcing particles, thereby altering the material’s response to external stimuli, such as mechanical and thermal energy, which are inherently dependent on the molecular dynamics of the system [40].

It is proposed that SiO_2_ nanoparticles, like other nanostructured reinforcements, act as high-energy sites within the polymeric matrix [39]. Through electrostatic interactions, primarily Van der Waals forces, these nanoparticles alter the mobility of the polymer chains by physically interacting with the surface hydroxyl (-OH) groups present on the SiO_2_ nanoparticles and the methyl groups distributed along the PDMS backbone [41]. Previous studies have demonstrated the absence of new chemical bonds or distinct configurations in PDMS–SiO_2_ composites [7], confirming that the interaction is predominantly physical rather than chemical. Since the interaction is electrostatic in nature, complete immobilization of the polymer chains is avoided, unlike what occurs in systems where covalent bonding restricts mobility. Such chemical interactions have been shown to lead to excessive rigidification and a marked reduction in the maximum strain achieved by the material [42]. In contrast, this physical interaction allows the reduction of polymer chain mobility while leading effective load distribution throughout the network, resulting in enhanced thermal and mechanical resistance, without compromising the elastomeric behavior of the PDMS matrix [28,37].

With the above mentioned, the incorporation of SiO_2_ nanoparticles with varying sizes and concentrations as reinforcing agents significantly influence the mechanical and thermal performance of the composites. A progressive increase in mechanical properties was observed with increasing nanoparticle content, reaching peak values at the highest reinforcement level (15%) in most of the studied groups. Furthermore, particle properties such as size and agglomeration were shown to directly affect the effective interfacial surface area available for interaction with the polymer chains [43], thereby also influencing the thermal and mechanical performance exhibited by the polymeric composites.

In 15-b and 10-b groups, a decline in ultimate strain and tensile stress was observed at the highest concentrations of reinforcing particles. This behavior has been associated with reinforcement agglomeration processes [20]. The reduction in mechanical performance is attributed to a decrease in the effective interaction area, as the aggregation of reinforcing particles leads to the formation of heterogeneous regions within the material. These regions result in stress concentration points and a subsequent reduction in mechanical strength [4,9].

Among the reinforcement systems used (SiO_2_-a and SiO_2_-b), the pH of the sol–gel medium directly influenced the final physicochemical properties of the synthesized particles [18]. Previous studies have demonstrated that SiO_2_ particles synthesized under alkaline conditions (SiO_2_-b) develop larger particle sizes and contain a higher concentration of by-products, such as TEOS, ethanol, and acetone, compared to SiO_2_-a [7]. These differences are attributed to distinct reaction pathways followed during the hydrolysis and condensation stages of the sol–gel process [18,19]. These nanoparticles exhibited greater thermal and mechanical properties in composites reinforced with SiO_2_-b (Table 1 and Table 2).

Although the increased surface area of SiO_2_-a particles might initially seem advantageous, the lower surface energy of SiO_2_-b particles potentially reduces agglomeration and promotes a higher degree of dispersion [44]. These factors contribute to a more uniform particle size and distribution throughout the composite [45].

Additionally, the higher presence of sol–gel by-products such as TEOS in SiO_2_-b [7] influenced the crosslinking conditions of the PDMS system after particle formation. An increased concentration of TEOS directly enhanced the final crosslinking degree, improving the mechanical properties of the polymer [30]. However, at high concentrations, this effect may also lead to excessive rigidity and reduced elasticity [22], as observed in the 15-5b and 10-15b composites.

The mechanical and thermal properties of the composites were enhanced by the formation of a more homogeneous system, which enabled a more uniform distribution of thermal and mechanical energy throughout the polymeric network [11]. Furthermore, an increased crosslinking degree contributed to an improvement in tensile strength [1], with the 5-15b composite exhibiting the most favorable thermal and mechanical performance among the evaluated samples.

The composites exhibiting the highest mechanical performance (5-15b and 10-15a) demonstrated variations in solvent absorption properties and crosslinking efficiency (Table 5). The reduced solvent absorption observed in the composites was attributed to the presence of SiO_2_ particles occupying the free spaces between polymer chains, thereby decreasing the intramolecular free space within the structure and consequently limiting the uptake of solvent molecules [46]. Additionally, the minor decrease in crosslinking efficiency (X_y_) may result from the higher concentration of unreacted by-products such as ethanol and TEOS, generated during hydrolysis and condensation reactions [19,47]. These small molecules were removed through solvent immersion during the gravimetric test.

The hydrophobicity and volumetric conductivity tests confirmed the absence of significant variations (either increase or decrease) in these essential insulation properties. The preservation of hydrophobic characteristics (>95°) is attributed to the ability of PDMS to form a surface layer upon deposition [5,32]. This phenomenon leads to the formation of a polymer-rich layer, maintaining hydrophobicity across composites with different reinforcer concentration and structure (SiO_2_-a and SiO_2_-b). The stability of volumetric resistivity in the reinforced composites is associated with the inherently insulating nature of silicon-based structures (SiO_2_ NPs and PDMS) that constitute the composites [1,43]. The use of these silicon composite systems ensures the retention of high electrical resistance values (~10^11^ Ω/cm) [24].

Like the insulation properties, adhesion was also maintained upon the addition of reinforcements (SiO_2_-a and SiO_2_-b), with all composites classified as A4 according to the ASTM D3359 standard [35]. This behavior is also attributed to the formation of an interfacial PDMS layer between the ceramic substrate and the composite [4,5], preventing adhesion from being significantly affected by the reinforcement concentration and type. In contrast, the PDMS:TEOS ratio appeared to induce a distinguishable change in adhesion among the control groups, which was associated with the crosslinking degree of PDMS [48].

The enhanced combination of mechanical reinforcement, hydrophobicity, and electrical insulation properties achieved in the PDMS-SiO_2_ composites highlights their potential for broader industrial applications beyond electrical insulation. In the aerospace sector, materials with high elasticity, low density, thermal stability, and environmental resistance are essential for components such as flexible coatings, sealants, and insulation layers in aircraft and spacecraft systems [49]. Likewise, in the biomedical field, reinforced PDMS composites are increasingly explored for the fabrication of polymeric prostheses and flexible implants, where mechanical modification, biocompatibility, and sustained surface hydrophobicity are critical to ensure long-term stability across different types of tissues [50]. Moreover, these composites show significant promise in emerging applications such as wearable electronic devices [51], and microfluidic systems [52], where simultaneous requirements of mechanical flexibility and chemical resistance are critical. Therefore, the development of SiO_2_-reinforced PDMS materials offers a versatile platform for advancing next-generation multifunctional devices across diverse technological fields.

## 4. Materials and Methods

### 4.1. Materials

For all synthesis and characterization processes conducted in this study, materials were washed with distilled water and dried to remove any contaminants or impurities. The precursors employed for the synthesis of the PDMS-%SiO_2_ composites included acetone (C_3_H_6_O) (99.5%, Meyer^®^, Mexico City, Mexico), hydroxy-terminated polydimethylsiloxane (PDMS-OH) (2550–3570 cSt, Sigma Aldrich^®^, Milwaukee, WI, USA), tetraethyl orthosilicate (TEOS) (98%, Sigma Aldrich^®^, Wuxi, China), ammonium hydroxide (NH_4_OH) (28%, J.T. Baker^®^, Madrid, Spain), nitric acid (HNO_3_) (65–70%, J.T. Baker^®^, Madrid, Spain), and dibutyltin dilaurate (DBTDL) (95%, Sigma Aldrich^®^, Milwaukee, WI, USA).

### 4.2. Synthesis of PDMS-SiO_2_ Nanocomposites

The in situ synthesis of PDMS-%SiO_2_ nanocomposites was conducted using the sol–gel method and the crosslinking reaction of the hydroxyl-terminated PDMS matrix, employing magnetic and ultrasonic stirring techniques, as described in previous investigations [4,7]. The process began with a solution of acetone and PDMS in a weight ratio of 1:0.6 (PDMS:C_3_H_6_O). The solution was stirred up and homogenized for 1 h. Subsequently, TEOS was introduced according to the desired nanoparticle concentration (0%, 5%, 10%, and 15% by weight). The reported particle concentrations were calculated based on the particle yield obtained during the SiO_2_ sol–gel synthesis.

The hydrolysis process and subsequent condensation of SiO_2_ nanoparticles were initiated by the dropwise addition of the alkaline or acidic catalyst (NH_4_OH or HNO_3_), adjusting the pH to 10 (alkaline) or 2 (acidic) [7]. The solution was stirred for 2 h and neutralized to pH 7. TEOS was added in accordance with the selected PDMS:TEOS weight ratios (5:1, 10:1, and 15:1), followed by stirring for 1 h. The solution was degassed in an ultrasonic bath for 15 min to remove any remaining air bubbles and finally stirred mechanically for 5 min.

Crosslinking was initiated by the dropwise addition of the DBTDL catalyst at a weight ratio of 1:0.02 (PDMS:DBTDL). The mixture was mechanically stirred for an additional 5 min, deposited and allowed to be cured at room temperature for 24 h.

### 4.3. Characterization Techniques

The synthesized composites were mechanically analyzed through tensile tests to select the most suitable study groups for subsequent thermal, electrical, and surface characterizations.

The tensile tests were performed in accordance with ISO 527-1 standards [53], using type 5B dog-bone specimens with five repetitions per group. A Shimadzu AGS-X universal testing machine equipped with a 100 N load cell, a crosshead speed of 30 mm/min, and rough-surface grips were employed. The applied force and displacement were recorded throughout the test until rupture, allowing the determination of the ultimate tensile stress, elastic modulus, and ultimate strain of the composites.

Thermal behavior of the composites was analyzed to evaluate the effect of SiO_2_ reinforcers on various energy-related processes within the PDMS matrix. Dynamic mechanical analysis (DMA) was conducted using a Perkin Elmer DMA 7 Dynamic Mechanical Analyzer, employing a heating ramp of 5 °C/min, a static force of 300 mN, a dynamic force of 250 mN, and a temperature range of −130 to 80 °C. Thermogravimetric analysis (TGA) was performed using a thermogravimetric analyzer with a heating ramp of 5 °C/min and a temperature range of 30–700 °C. This thermal characterization provided deeper insights into the nature of the interaction between the polymeric matrix and the reinforcements.

Once the composites with the best mechanical performance were selected, their crosslinking efficiency, hydrophobicity, conductivity, and adhesion properties were assessed to ensure that these intrinsic and surface characteristics were not adversely affected.

Crosslinking efficiency (*X_y_*) was evaluated using gravimetric methods, determining the material loss when dispersed in an ethanol solution by triplicate [54]. Hydrophobicity tests were conducted through contact angle measurements using a Ramé-hart goniometer model 250-U1 with an average water droplet volume of 8.43 µL. Volumetric conductivity was measured using a Keithley 6517B electrometer equipped with an 8009 fixture for high resistivity. Samples with dimensions of 6 × 7 cm and a thickness of 0.7 mm were evaluated using the reverse voltage method for high-resistance measurements, with a sweep range of -250 to 250 V and a current of 20 mA [33]. Finally, surface adhesion was assessed according to the ASTM D3359 standard using the cross-cut tape test method [35]. Material was deposited on a ceramic substrate with an area of 15 cm^2^ and an average thickness of 0.5 mm. Each composite was tested in triplicate and categorized on a scale from 0 A to 5 A.

## 5. Conclusions

The in situ sol–gel synthesis of PDMS-SiO_2_ composites enabled the fabrication of advanced insulating materials with significantly enhanced mechanical and thermal performance. The study demonstrated that the concentration of SiO_2_ nanoparticles, the pH medium of the sol–gel process (acidic or alkaline), and the PDMS:TEOS ratio are key factors that modify the physicochemical properties of the composites. Among the reinforced polymers, those incorporating alkaline-synthesized SiO_2_ (SiO_2_-b) exhibited superior mechanical and thermal properties, particularly the 5-15b group, which achieved the highest fracture stress and viscoelastic modulus. This improved behavior was attributed to a more homogeneous size distribution, lower surface energy, and better dispersion of the nanoparticles, which minimized agglomeration and facilitated effective stress transfer within the polymer network. Additionally, maintaining essential surface properties (hydrophobicity, electrical resistivity, and adhesion) confirmed that the in situ incorporation of SiO_2_ did not compromise the functional performance of the coatings. These findings supported the implementation of in situ sol–gel synthesis under alkaline catalysis as an effective strategy for the development of PDMS-based nanocomposites with enhanced mechanical and thermal performance for high-demand insulation applications.

Moreover, the in situ approach facilitated the incorporation of nanostructured reinforcements by enabling the identification and control of key experimental variables for the development of next-generation composites. This synthesis strategy provided a versatile platform for the tailored modification of polymeric matrices, thereby broadening the scope of application of these materials both in advanced technological and industrial fields.

## Figures and Tables

**Table 1:** Tensile mechanical test properties for PDMS-%SiO_2_ composites.

	Elastic Modulus (MPa)					
PDMS:TEOS	15		10		5	
SiO_2_^1^ (%w)	a	b	a	b	a	b
0	0.39 ± 0.01	0.39 ± 0.01	0.39 ± 0.01	0.39 ± 0.01	0.50 ± 0.013	0.50 ± 0.01
5	0.46 ± 0.02	0.47 ± 0.004	0.51 ± 0.03	0.48 ± 0.02	0.49 ± 0.010	0.48 ± 0.01
10	0.50 ± 0.001	0.50 ± 0.01	0.52 ± 0.02	0.51 ± 0.01	0.50 ± 0.009	0.52 ± 0.01
15	0.56 ± 0.01	0.46 ± 0.01	0.54 ± 0.03	0.59 ± 0.01	0.52 ± 0.023	0.60 ± 0.02
	**Ultimate Strain (MPa)**					
PDMS:TEOS	15		10		5	
SiO_2_^1^ (%w)	a	b	a	b	a	b
0	3.00 ± 0.15	3.00 ± 0.15	2.12 ± 0.26	2.12 ± 0.26	2.87 ± 0.54	2.87 ± 0.54
5	1.70 ± 0.35	3.11 ± 0.45	1.88 ± 0.32	3.25 ± 0.46	3.00 ± 0.18	3.48 ± 0.45
10	2.91 ± 0.24	3.80 ± 0.31	3.16 ± 0.13	3.48 ± 0.38	3.52 ± 0.32	2.76 ± 0.19
15	2.79 ± 0.28	2.91 ± 0.05	3.88 ± 0.56	2.46 ± 0.33	3.40 ± 0.35	4.08 ± 0.19
SiO_2_^1^ (%w)	**Ultimate Tensile Stress (MPa)**					
PDMS:TEOS	15		10		5	
[C] SiO_2_^1^	a	b	a	b	a	b
0	0.59 ± 0.07	0.59 ± 0.07	0.47 ± 0.04	0.47 ± 0.04	0.73 ± 0.02	0.73 ± 0.03
5	0.48 ± 0.04	0.75 ± 0.09	0.57 ± 0.05	0.85 ± 0.14	0.82 ± 0.09	0.90 ± 0.11
10	0.93 ± 0.11	1.24 ± 0.13	1.15 ± 0.03	1.28 ± 0.23	1.28 ± 0.10	1.00 ± 0.11
15	1.34 ± 0.22	0.76 ± 0.05	1.94 ± 0.22	1.34 ± 0.27	1.53 ± 0.30	2.71 ± 0.16

^1^ Rows correspond to the SiO_2_ concentration, and columns correspond to the catalyst type: acidic (a) or alkaline (b).

**Table 2:** Dynamic mechanical analysis test results for PDMS-%SiO_2_ composites.

	G″ (MPa)	G′ (MPa)	Tan δ	Tg (°C) ^1^
15-0	0.035	0.970	0.036	−66.79
15-5a	0.013	1.508	0.008	−70.14
15-10a	0.058	1.080	0.053	−60.72
15-15a	0.086	1.521	0.057	−59.74
15-5b	0.062	1.014	0.061	−59.81
15-10b	0.063	1.160	0.054	−59.88
15-15b	0.014	1.267	0.011	−72.72
10-0	0.064	1.097	0.059	−69.31
10-5a	0.064	1.191	0.053	−79.25
10-10a	0.063	1.103	0.057	−66.60
10-15a	0.062	1.161	0.054	−67.00
10-5b	0.064	1.059	0.060	−61.36
10-10b	0.071	1.101	0.057	−61.15
10-15b	0.071	1.301	0.053	−61.05
5-0	0.058	0.972	0.060	−61.77
5-5a	0.060	1.035	0.058	−65.21
5-10a	0.068	1.081	0.063	−64.31
5-15a	0.076	1.174	0.064	−60.27
5-5b	0.060	1.246	0.048	−63.34
5-10b	0.097	1.267	0.077	−62.34
5-15b	0.077	1.398	0.055	−52.54

^1^ T_g_ was calculated by extrapolating the first mechanical transformation process from the G′ vs. T.

**Table 3:** Thermogravimetric analysis for PDMS-%SiO_2_ composites.

Composite	Intersection (100 °C)	Max. Mass Loss		Reisdual Mass 700 °C (%)
		T (°C)	Rate (%/°C)	
15-0	327	392	0.48	0
10-0	351	419	0.79	0
10-5a	391	447	0.72	1.17
10-10a	395	437	0.83	2.26
10-15a	393	425	0.68	2.94
5-0	358	435	0.65	2.27
5-5b	377	445	0.86	2.85
5-10b	405	451	0.58	8.35
5-15b	447	550	0.5	16.16

**Table 4:** Gravimetric test results for crosslinking efficiency on PDMS-SiO_2_ selected composites.

Group	A (%)	$L_{w}$ (%)	$X_{y}$ (%)
15-0	3.65 ± 0.55	3.59 ± 1.04	96.41 ± 1.04
10-0	2.15 ± 0.81	1.71 ± 0.86	98.29 ± 0.86
5-0	2.23 ± 0.90	1.78 ± 0.97	98.22 ± 0.97
10-15a	1.12 ± 0.80	3.49 ± 0.34	96.51 ± 0.34
5-15b	0.98 ± 0.75	3.28 ± 0.64	96.72 ±0.64

**Table 5:** Water contact angle and volumetric resistivity results for PDMS-SiO_2_ selected composites.

Group	Contact Angle (°)	Resistivity (10^11^ Ω/cm)
15-0	100.4 ± 2.1	3.6 ± 0.6
10-0	99.1 ± 1.6	3.1 ± 0.8
5-0	99.7 ± 5.0	2.5 ± 0.9
10-15a	99.3 ± 2.3	2.6 ± 0.6
5-15b	98.1 ± 3.8	2.4 ± 0.6

## Data Availability

Data are contained within the article.

## Abbreviations

The following abbreviations are used in this manuscript:

DBTDL	Dibutyltin dilaurate
DMA	Dynamic mechanical analysis
PDMS	Polydimethylsiloxane
TEOS	Tetraethyl orthosilicate
TGA	Thermogravimetric analysis
